# Detection of Single Ag Nanoparticles Using Laser Desorption/Ionization Mass Spectrometry

**DOI:** 10.1021/jasms.3c00137

**Published:** 2023-06-12

**Authors:** Michal Žalud, Vadym Prysiazhnyi, Antonín Bednařík, Jan Preisler

**Affiliations:** Department of Chemistry, Faculty of Science, Masaryk University, 625 00 Brno, Czech Republic

## Abstract

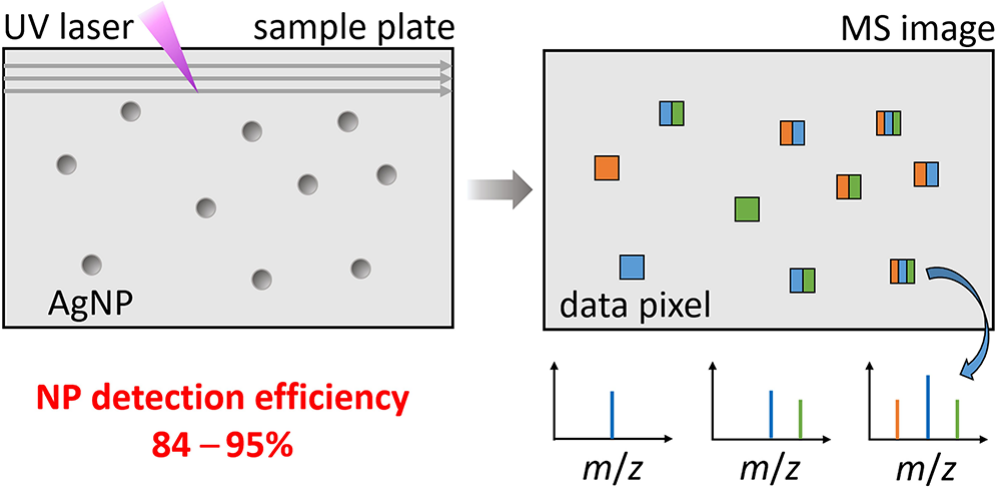

The detection of a single entity (molecule, cell, particle, etc.) was always a challenging subject. Here we demonstrate the detection of single Ag nanoparticles (NPs) using subatmospheric pressure laser desorption/ionization mass spectrometry (LDI MS). The sample preparation, measurement conditions, generated ions, and limiting experimental factors are discussed here. We detected from 84 to 95% of the deposited 80 nm Ag NPs. The presented LDI MS platform is an alternative to laser ablation inductively coupled plasma mass spectrometry for imaging distribution of individual NPs across the sample surface and has a great potential for multiplexed mapping of low-abundance biomarkers in tissues.

## Introduction

With the rapid development of nanoscience and nanotechnology, nanoparticles (NPs) have become broadly used in sensing, imaging, drug delivery, or therapeutics, especially in biomedicine.^1−3^ Nowadays, more and more emphasis is placed on detecting a single small nanoscale object, i.e., a molecule^4^ or nanoparticle.^5,6^ Single-molecule or single-particle (SP) analysis belongs to the most challenging subjects in analytical nanoscience, whether it is the measurement of the concentration of particles/molecules in solution or the detection of single particles on the solid sample surface. Therefore, new methods suitable for this purpose are being developed, and new approaches for tracking and imaging NPs in different systems and environments are needed.

Many methods for analyzing NPs have been developed already. The most widely used are scanning electron microscopy (SEM),^12^ which measures a signal produced by incident or secondary electrons, and transmission electron microscopy (TEM),^12^ which measures the change of electron energy passing through the sample. These methods produce high-resolution images, making them suitable for SP analysis, but they lack in-depth information about the chemical and molecular structure, not to mention many limitations in sample preparation and sample types.

Mass spectrometry (MS) allows direct measurement of the mass-to-charge ratio (*m*/*z*) of ions generated from NPs. Single-particle inductively coupled plasma mass spectrometry (SP ICP MS) has become a routine technique for the detection, determination, and elemental characterization of NPs. This method offers NP analysis in aerosols, suspensions, and solid matrices.^5^ In particular, laser ablation (LA) SP ICP MS can be used to image nanoparticles on the sample surface or in samples.^16−19^ Conventional UV laser ablation leads to breakdown of NPs desorbed from the sample surface already in the ablation cell, which makes effective NP counting in the SP ICP MS mode difficult. The use of a 2940 nm IR laser for LA ICP MS eliminates the disintegration of NPs due to their low absorption in the IR.^20^ For the SP MS analysis, developing an imaging method with merits like high spatial resolution, better quantitative credibility, and the capability to provide complex chemical information about the sample is desirable.

The laser desorption/ionization (LDI) MS technique has been tightly connected to nanomaterials for decades since Tanaka used cobalt nanopowder to enhance the signal of large biomolecules.^21^ This technique can be employed in mass spectrometry imaging (MSI) mode as an effective tool for mapping a wide range of analytes, primarily focusing on small molecules and biomolecules.^22^ Currently, the most frequently employed mode of LDI is matrix-assisted laser desorption/ionization (MALDI), taking advantage of organic matrices, which absorb the laser energy and efficiently ionize analyzed molecules. The use of nanostructures, particularly NPs, resulted in the development of the so-called surface-assisted LDI MS (SALDI MS). Using metal NPs as a matrix that adsorbs laser irradiation and assists in both desorption and ionization improved the detection of tumor markers of the cell membrane,^23^ analysis of metabolites,^24^ or lipids.^25,26^ However, NPs were typically used as a matrix here; they were not an object of investigation. In the LDI studies mapping NP distribution, NPs were present in significant excess in the samples, producing abundant metal ions and adducts.^24,27,28^ Also, direct LDI MS detection of whole small Au clusters with sizes up to a few nm (8–29 kDa) modified by different *n*-alkanethiolate ligands was reported.^29,30^ Au NPs were utilized as mass tags to detect proteins via an immunocapture laser ionization MS assay.^31^ Moreover, the multiplexed detection of molecular ligands attached to nanoparticles was reported with a limit of detection down to attomoles.^1,32^ Current MALDI mass spectrometers allow imaging with spatial resolution down to micrometers. The use of ultra-high-mass resolution Fourier transform mass spectrometers facilitates the identification of species of interest and their differentiation from noise components. These features, together with the increasing sensitivity of modern instruments, further promote LDI MSI as a promising platform for SP analysis. Recently, we used a subatmospheric pressure (subAP) dual MALDI/ESI source interfaced to an orbital trap for imaging of Au NPs; signals were detected from as low as ten 50 nm AuNPs.^33^ Furthermore, the technique has a great potential for multiplexed SP detection of NPs with different elemental compositions and simultaneous detection of various ligands attached to the NP surface.^34^

Here we report the LDI MSI method for single-NP detection employing specific gas-phase reactions in a subAP ion source connected to an orbital trapping MS. Special attention is paid to sample preparation to achieve deposition of separated individual NPs over a predefined area. The data processing is essential, showing potential difficulties and uncertainty of evaluation, which leads to different numbers of detected NPs in the prepared samples. Based on the results, the limiting factors affecting the detection possibilities of single NPs in subAP LDI MSI are explored.

## Experimental Section

### Chemicals

A Ag NP suspension was purchased from nanoComposix (San Diego, CA). The as-received Ag NPs were 80 ± 3 nm in diameter and had a 8.1·10^9^ NP/mL concentration (provided by the manufacturer). Ultrapure water was prepared by a Direct-Q 3UV Millipore water purification system (Merck, Germany). Ethanol (99.8%) and methanol (99.8%) were purchased from Merck (Germany). Si wafers (p-type, (111) orientation, <20 Ω·cm, ScienceSevices, Germany) were cut into smaller pieces (typically 2 × 3 cm), cleaned with methanol, and carefully wiped by a lint-free tissue. Glass substrates were half of a standard microscope slide (37.5 mm (*L*) by 25 mm (*W*)) that fit into the ion source sample holder. Before NP deposition, those were cleaned with ethanol and dried by a flow of compressed air.

### Sample Preparation and Characterization

Reference Ag films were made on a microscope slide using a laboratory-built magnetron sputtering device in a vacuum chamber provided by Activair, Czech Republic. In this device, the vacuum pumping system consisted of rotary and turbomolecular pumps, giving <10^–7^ Torr base pressure. The 3″ TORUS magnetron gun (Kurt J. Lesker, USA) with a Ag target (99.99% pure, Camex s.r.o., Czech Republic) was operated at 11 mTorr pressure in Ar (99.996% pure) at 50 W DC power supplied by a PD500X high-voltage power supply (Kurt J. Lesker, USA). The deposition speed of Ag at the given conditions was 8.6 nm/min, as determined by profilometer DektakXT (Bruker, USA). Films with a thickness of 20 nm were prepared by sputtering for 140 s.

The samples with individual Ag NPs scattered across the surface were prepared using an MJ-ABP-01-50 DLC drop-on-demand piezoelectric dispenser (MicroFab Inc., USA). The piezoelectric dispenser was attached stationary approximately 1 mm above the microscope glass slide fixed on an *XY* positioning table moved by 7T175-series micrometric stages (Standa, Lithuania). During a single voltage pulse, a 65 pL droplet of Ag NP suspension emitted from a nozzle of the dispenser was deposited on a defined position on the glass slide. Therefore, the concentration of NPs in the utilized suspension and the number of generated droplets defined the number of deposited NPs. Additional details about the used equipment can be found in Section 1 of the Supporting Information.

A single sample for MS imaging consisted of an array composed of 10 × 5 spots with a spacing of 300 μm in both the *X* and *Y* directions, resulting in a 2.7 × 1.2 mm area (see Section 2 in the Supporting Information with examples of SEM images obtained from two arrays on a Si substrate). The Ag NP suspension was diluted to contain ∼5 NPs per 65 pL based on the NP concentration provided by the producer; therefore, a total of ∼250 NPs was expected in each spot array.

The number of Ag NPs deposited on the surface was verified and counted using Versa 3D SEM (FEI, USA). Here, pieces of Si wafer were used as substrate; therefore, NP distribution could be measured by SEM directly. The NP suspension was deposited on the Si substrate right before and after the deposition on the glass slide to keep constant experimental conditions and eliminate possible effects of suspension aging. The SEM signal was acquired from secondary electrons collected using the Everhart-Thornley detector, an acceleration voltage of primary electron beam of 10 kV, and a beam current of 83 pA.

### SubAP LDI MS/MSI

**Figure 1:**
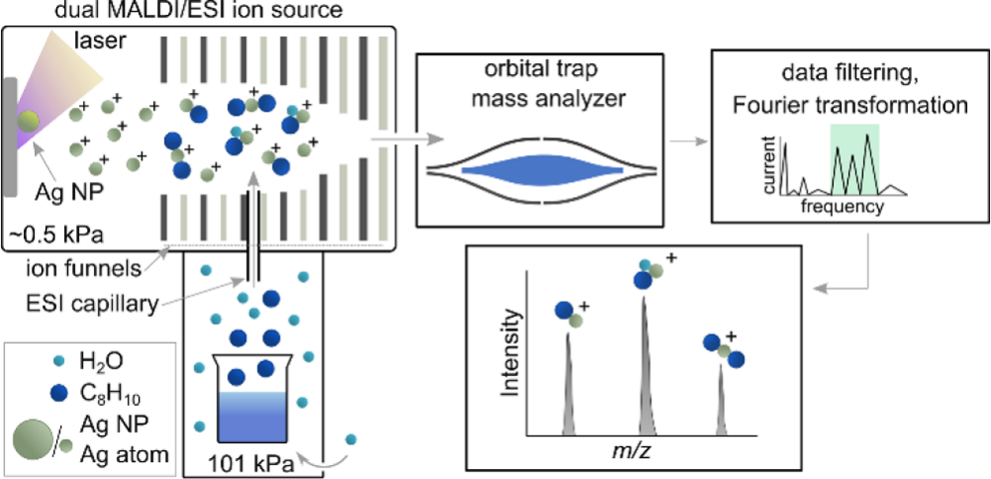
Schema of experiment with dual MALDI/ESI ion source interfaced to orbital trap.

The ions were produced inside a dual MALDI/ESI subatmospheric ion source (subAP/MALDI (ng), MassTech Inc., USA). The MALDI part was equipped with a 355 nm Nd:YAG laser with energy <1 μJ/pulse, and the sample was attached to an *XY* stage. The ESI inlet was used to infuse xylene vapors through a 125 mm long capillary tube with a 0.4 mm inner diameter for gas-phase reactions with Ag^+^ ions enhancing the ion signal. It was achieved by placing a 20 mL beaker with 5 mL of liquid xylene in front of the ESI capillary inlet. The signal enhancement due to xylene vapors is discussed in Section 3, Supporting Information. The subAP ion source operated at ∼3.8 Torr pressure was connected to a Q Exactive Plus Orbitrap mass spectrometer (Thermo Fisher Scientific Inc., Germany) via a double-stage ion funnel. The experimental arrangement of the dual MALDI/ESI ion source is presented in Figure 1.

The MS and MSI measurements were done under the following conditions: resolving power 35 000 (at *m*/*z* 200), C-trap ion injection time (*IT*) 239 ms, *m*/*z* measurement range from 50 to 750 Da, laser pulse frequency 1 kHz, positively charged ion collection, pixel size 10 × 10 μm (for MSI measurements). The sample was irradiated in the constant speed raster motion mode, which means the sample plate was moving while the laser was constantly shooting. The sample plate velocity was set to 0.0382 mm/s, thus defining the time spent on a pixel 261 ms. This includes the *IT* needed to collect ions in the C-trap (239 ms) and the time to inject ions from the C-trap to the orbital trap (22 ms). The MSI runs were set up with no C-trap charge detector prescan; therefore, independently of a number of ions, the *IT* value was fixed for each pixel. A PM160 optical power meter (Thorlabs, USA) was used to measure laser energy. The sensor was placed between the last focusing lens and the sample plate. The laser power was determined as the difference between a background signal with the laser turned off averaged over at least 20 s (≥200 measurement points) and a signal with the laser on averaged over at least 40 s (≥400 measurement points). Energy per pulse values were calculated from the experimental values of the laser power. The laser spot size was estimated from an ablated area of a thin film irradiated by laser for 0.4 s. Based on the used laser energy, the spot size ranged from 10.2 × 15.8 μm to 11.5 × 17.9 μm.

## Data Processing

The data was processed using the open-source MSI Reader package^35^ and self-developed Python scripts. The noise level was determined from RAW data using an Xcalibur Qual Browser provided in a package from the instrument manufacturer. Ion signals at a designated *m*/*z* with a 0.005 Da window were extracted from the RAW data files. Then, pixels with ion signal values above the noise level were analyzed and subjected to statistical analysis. Note that noise here slightly differs from that in a typical mass spectrum. Due to large RAW data file sizes at high resolution, most noise data is filtered out to save space (done routinely as a part of data processing by manufacturer’s software). Nevertheless, a noise at random *m*/*z* is still present in mass spectra alongside measured signals. The term “noise level” used below is defined as the intensity of those randomly distributed signals. It is evident that the noise value depends on the experimental parameters; it was 420 ion counts for the measurement settings used in this work. Because the number of these randomly distributed noise data points is much lower than that of all data points in the *m*/*z* range, a signal with an intensity equal to or higher than 420 ion counts at the monitored *m*/*z* indicates the presence of the ion of interest.

The ions of interest (Ag^+^, [Ag + C_8_H_10_]^+^, [Ag + C_8_H_10_ + H_2_O]^+^, and [Ag + 2(C_8_H_10_)]^+^) have isotopic patterns with the two most intense components being ^107^Ag and ^109^Ag. Two approaches used to determine whether a diagnostic ion is present in a pixel are explained on the example of [Ag + C_8_H_10_]^+^ ions with the two most intense components at *m*/*z* 212.983 and 214.983, respectively:

**Approach I**
1) The pixel is considered as a data pixel only when both isotopic components of a specie, for example, [Ag + C_8_H_10_]^+^ at *m*/*z* 212.983 and 214.983 exceed the noise level.
2) Summed [Ag + C_8_H_10_]^+^ ion signal intensities of the two isotopic components from a single pixel are used for evaluation. Because the threshold for the intensity of each isotopic component is 420 ion counts, the minimum summed ion signal intensity of the two isotopic components is 840 ion counts.

**Approach II**
1) The pixel is considered as a data pixel if the ion signal of at least one of the isotopic components of a specie, for example, [Ag + C_8_H_10_]^+^ at *m*/*z* 212.983 or 214.983 exceeds the noise level of 420 ion counts.
2) The [Ag + C_8_H_10_]^+^ ion signal intensity for a single pixel is counted as the sum of the intensities of both isotope components (same as in Approach I).

The reason for introducing Approach II was the observed variation in the ratio between isotopic components at low ion signal intensities. The ion detection in the Orbitrap does not provide for precise isotopic ratio measurements. The ratio between the peak heights of the isotopic component experimentally determined from 20 nm Ag film (averaged from 400 spectra at 1000 ms injection time) was 1.00 ± 0.02, significantly deviating from the theoretical ratio of 1.07 (taken as a ratio of relative mass fraction of 47.51 and 44.14% from the mass calculator.^36^ Yet, the experimentally determined ratio varied in a much broader range in the case of low-intensity signals, i.e., signals of individual NPs. Within the measured data set of 622 pixels generating [Ag + C_8_H_10_]^+^, the ratio was 1.15 ± 0.41. Therefore, an isotopic component of ion signals can be missing in the mass spectra in the case of ions with intensities close to the noise level because software can falsely attribute low-intensity signals to noise.^37^ Note that we did not have any control of the internal data processing and did not modify it.

Thus, Approach I is a conservative estimate, providing lower nanoparticle counts because part of the data can be lost, while Approach II yields higher nanoparticle counts due to the possible counting of false positives. The majority of presented data was treated by Approach I, but final percentages of detected NPs were recounted using both Approaches as the actual value probably lies between them.

## Results and Discussion

### Spot Array Sample for subAP LDI MSI

Exact numbers of NPs in spot arrays prepared by the piezoelectric dispenser were counted using SEM. While SEM has more than enough capability to resolve each 80 nm Ag NP, it has a limited field of view: a single measurement area of 80 × 60 μm is the limit to resolve individual NPs in this case. It is defined by the pixel size, which can be calculated from microscope magnification and output image size (maximum image size is 6144 × 4415 pixels for the used microscope). Ideally, a single NP should be displayed on at least 5 × 5 pixels, i.e., 16 nm or less per pixel. A single droplet from the piezoelectric dispenser produced a spot with ∼300 μm diameter on glass, requiring the acquisition of many images and stitching them together to get a complete image of the spot. This would make counting the NPs a very time-consuming process with considerable instrumental costs. Moreover, the glass should be overcoated with a conductive layer, further reducing SEM sensitivity and incompatibility with the subsequent subAP LDI MSI experiments. Conversely, when a single droplet is deposited onto the Si substrate, a spot with a diameter of ≤20 μm is typically formed. Spots on Si substrates can be measured using SEM on as-deposited samples directly without coating and the necessity of image stitching (including the measurement of the droplet surroundings) to make sure that all NPs are counted. To quantify the NP number on the glass substrates, additional Si substrates for the preparation of spot arrays in the same batch were placed before and after the glass slides. The NP numbers on the Si substrates were averaged, yielding the expected number of Ag NPs deposited on the glass substrates.

**Figure 2:**
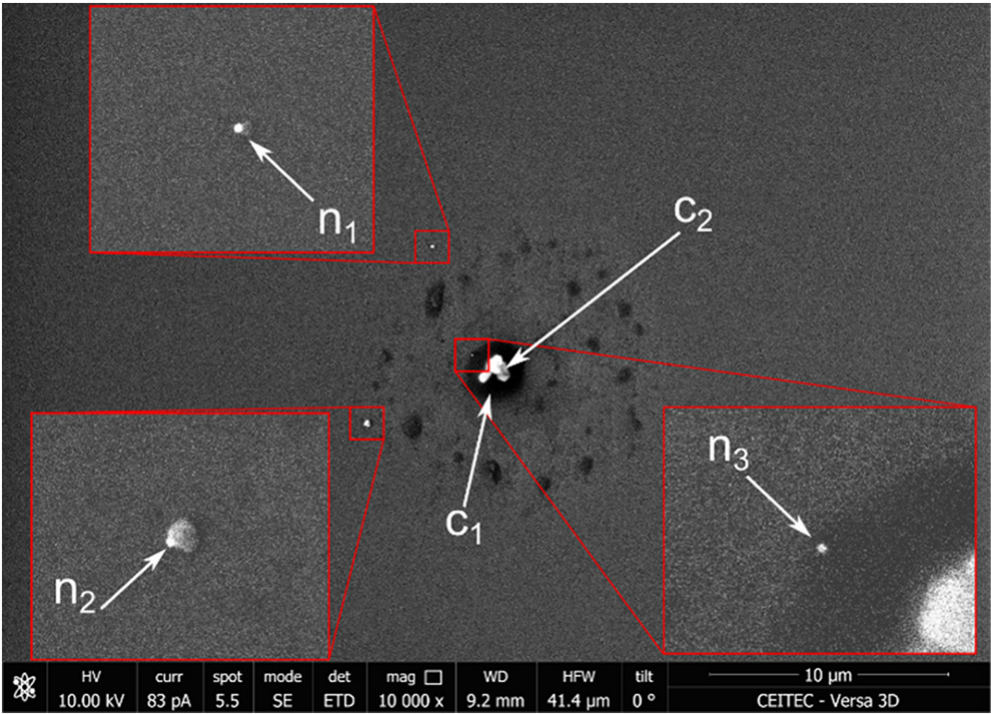
An SEM image of a single spot from the spot array showing two types of additive deposits (*c*_1_ – amorphous organic layer and *c*_2_ – nonconductive citrate crystals) and three typical positions of NPs (*n*_1_ – on Si substrate, *n*_2_ – attached to amorphous salt agglomerate, and *n*_3_ – embedded into the amorphous organic layer).

Figure 2 shows one of the spots on the Si substrate containing three Ag NPs. The presence of additives resulted in two distinct features in SEM: darker areas (position *c*_1_), representing an amorphous organic layer that can be a few tens of nanometers thick, and nonconductive citrate crystals (position *c*_2_), which were charged by the incident electron beam and gave a false-positive contrast. NPs were found in three positions: on the Si substrate (position *n*_1_), located on larger objects, probably consisting of amorphous citrate agglomerate (position *n*_2_), and embedded into the amorphous organic layer (position *n*_3_). The counting required special attention for NPs in positions *n*_2_ and *n*_3_. Careful NP counting on the control spot arrays gave 308 and 330 NPs on two arrays before and 298 and 300 on two arrays after glass slides. The number of Ag NPs in the standard sample for subAP LDI MSI was estimated to be 308 ± 15.

Another critical issue for subAP LDI MSI and correct NP counting is whether two NPs can be located in the same pixel (the pixel size was 10 × 10 μm) and the probability of an NP being just between two adjacent pixels. Thus, diagnostic ions would be generated from a single NP on both pixels. The probability of occurrence of two or more particles in the same pixel was estimated based on the Poisson distribution of NPs in the deposited spot array. For the used sample and pixel size, the signal from 308 NPs should be ideally detected in 307 pixels with two NPs on the same pixel only in one case. The probability of a Ag NP being located just between two adjacent pixels was estimated to be 0.4% by simple geometric approximation, meaning that in the prepared sample, the signal of only 1 NP contributes statistically to two adjacent pixels. The details about these estimations can be found in Section 4, Supporting Information.

Based on these estimates, it is safe to assume that each data pixel with a measured signal will be counted as a single NP. The negative error of nanoparticle detection efficiency introduced by this simplification does not exceed 1%.

### Mass Spectra from Individual Ag NPs

**Figure 3:**
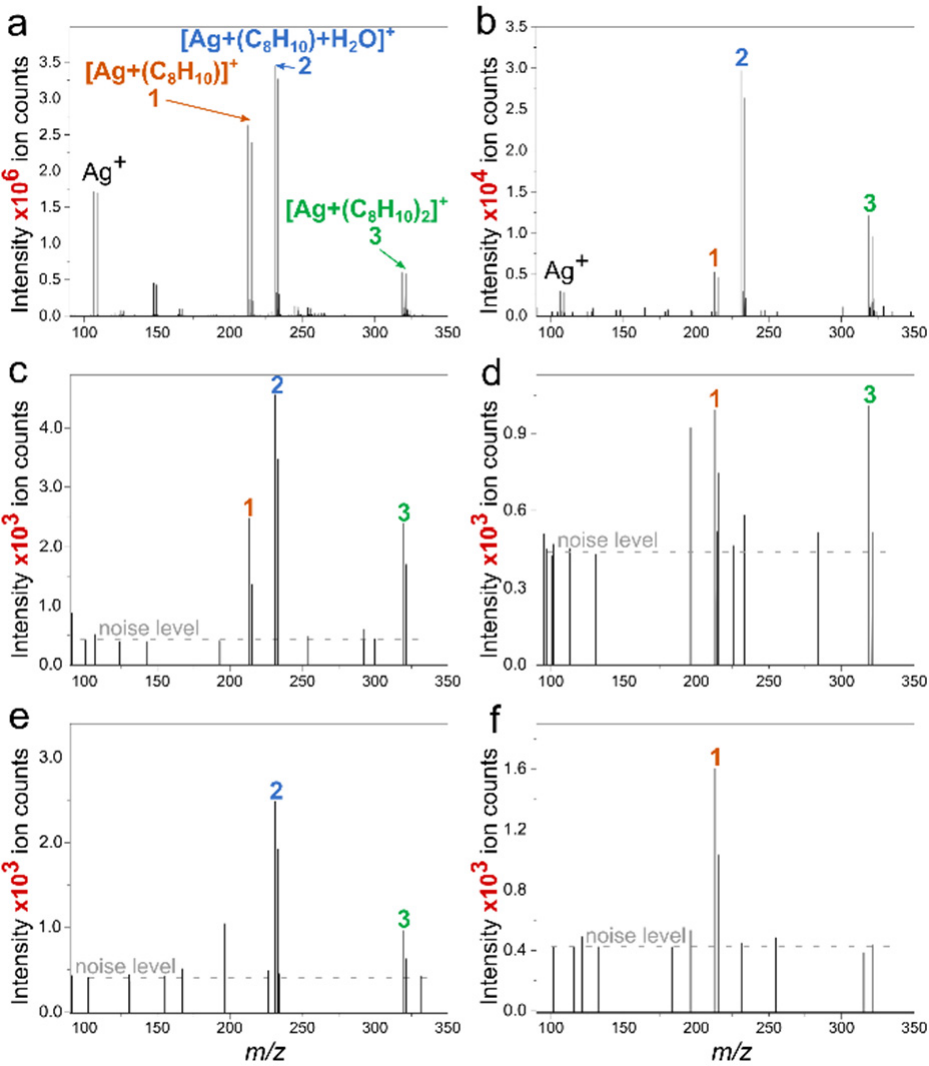
Mass spectra of Ag nanostructures measured in the subAP LDI MS with xylene vapor influx: (a) 20 nm magnetron-sputtered film; (b) the highest-intensity single-pixel mass spectrum measured from a sample spot containing ∼100 NPs; (c–f) examples of mass spectra from pixels containing a single NP.

The quality of mass spectra (the signal intensity, number, and relative ratios of the detected ions) was found to depend strongly on the quantity of silver. The mass spectrum of the sample with abundant Ag, represented by 20 nm magnetron-sputtered Ag film (averaged from 150 spectra, Figure 3a), contains the four most intense ions: Ag^+^ (*m*/*z* 106.905 and 108.905), [Ag + C_8_H_10_]^+^ (*m*/*z* 212.983 and 214.983; marked as **1**), [Ag + C_8_H_10_ + H_2_O]^+^ (*m*/*z* 230.994 and 232.994; marked as **2**), and [Ag + 2(C_8_H_10_)]^+^ (*m*/*z* 319.061 and 321.061; marked as **3**). Note that the ratio between the xylene-containing adduct ions is constant (verified by measurements on different days and using different Ag films). The three xylene-containing charged adducts were taken as the markers of NP presence in a particular pixel. Though the Ag^+^ ion was included in the calculations, it shall be omitted in the discussion for two reasons. (1) There were no pixels with an exclusive presence of Ag^+^ when data were treated by Approach I. When the MSI data were processed using Approach II, the Ag^+^ ions were found in the mass spectra almost exclusively together with the diagnostic ion **2**. (2) The intensity of Ag^+^ signals was an order of magnitude lower than the highest xylene-containing adducts. Moreover, the intensity ratio of diagnostic ions **1**, **2**, and **3** in the samples with a low number of NPs was not stable, as mentioned in the description of Approach II in the Data Processing section. Typically, but not exclusively, the intensity of diagnostic ions 1 and 3 was lower compared to ion 2 in the spectra of individual NPs. The differences between the detected ion composition of Ag film and individual NPs are due to the high gas-phase selectivity of the adduct formation, a different number of Ag atoms/ions ablated from the Ag surface, and the limited sensitivity of the mass analyzer.

Note that the *Y*-axis scale in the obtained spectra is in ion counts, arbitrary units resulting from Fourier transformation, filtering, and further signal processing of the transient signal of amplified ion current obtained from the Orbitrap electrodes.^38,39^Figure 3b shows a mass spectrum obtained from a pixel (10 × 10 μm) with the highest intensity of diagnostic ions recorded from a sample spot (∼300 μm) containing about 100 NPs (based on SEM), which were detected on multiple neighbor pixels. The type of detected ions remains the same, but the signal decreased by 2 orders of magnitude compared to the 20 nm Ag film; also, the ratio between ions **1**, **2**, and **3** is different and varies from pixel to pixel in this case. Figure 3c–f shows mass spectra extracted from pixels containing signal(s) of diagnostic ions in a dried spot with 1 NP/pixel (based on the probability calculations). The intensity dropped by 3 orders of magnitude compared to that for 20 nm Ag film, and the signals are close to the instrument noise, which was 420 ion counts for the MSI experiment conditions used in this work.

The experimentally measured isotopic ratio of Ag and Ag-containing ions (that should be 51.84:48.16 for ^107^Ag:^109^Ag) varied in a broader range (briefly discussed in the Data Processing section). It can stem from either a lower ion count or a negative impact of mathematical data processing of transient data. Figure 3d,e signifies that data processing is essential for counting pixels containing nonzero signals from diagnostic ions. In some extreme cases, only a single characteristic isotope peak of the Ag–xylene adduct was present, raising a valid question of whether data from these pixels should be used. Approach II addresses this issue as described in the Data Processing section.

### Mass Spectrometry Imaging of Individual Ag NPs

SubAP LDI MSI experiments were carried out at three laser energy levels: 0.31, 0.41, and 0.55 μJ/pulse; the laser frequency was 1 kHz. The estimated value of laser irradiation absorbed by an NP can be found in Section 5, Supporting Information; it shows it is not considerably different from the ratio of the used laser energy.

**Figure 4:**
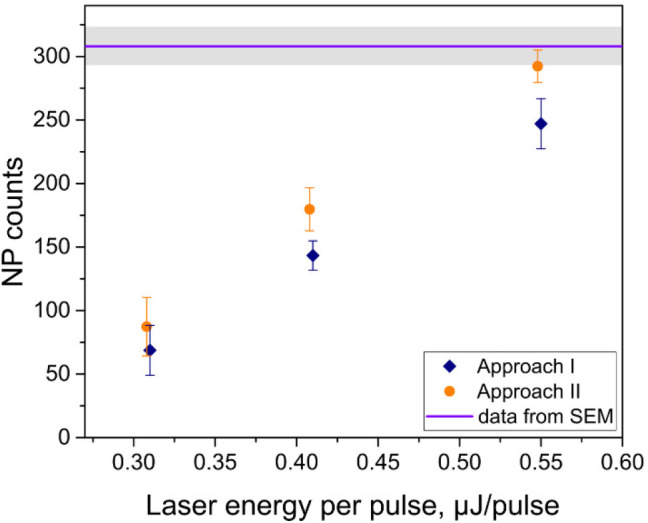
Detected NP counts (data pixels containing signals of diagnostic ions) as a function of laser energy. Data markers were intentionally shifted apart horizontally to prevent the overlapping of error bars. The upper line and band show the expected NP count and SD determined by SEM, i.e., 308 ± 15 NPs.

Figure 4 shows the number of detected NPs (i.e., the number of data pixels containing signals of diagnostic ions) evaluated by the two discussed Approaches as a function of laser energy. The NP numbers were obtained from three independently measured sample spot arrays. The detection efficiency of individual NPs by subAP LDI MSI increased with the increasing laser energy. Up to 84 or 95% of deposited NPs was detected at the highest energy, 0.55 μJ/pulse, as evaluated by Approaches I or II, respectively. The detection efficiency overcomes that of nebulizer SP ICP MS, typically ranging between 2 and 10%.^40^

**Figure 5:**
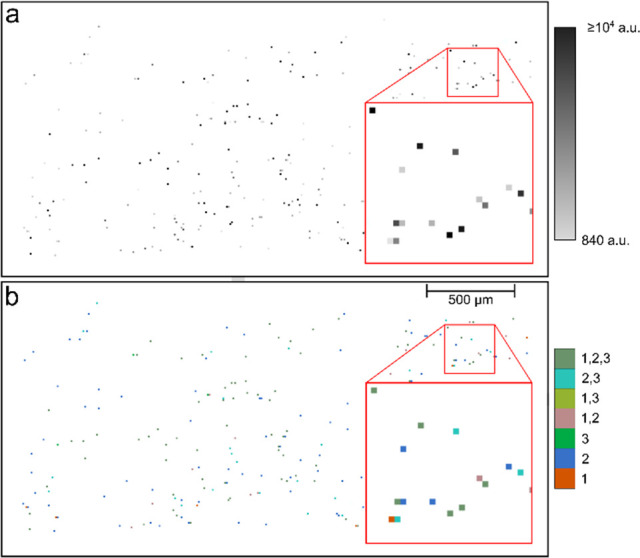
Example of MSI of a spot array with 308 Ag NPs irradiated at 0.55 μJ/pulse laser energy with 263 data pixels containing data with (a) the intensity scale, where a black pixel signifies intensity over 10 000 and lower ones are linear shades of gray; (b) color-coded data showing the specific diagnostic ions and their contribution.

An example of MSI data, where the detection of 84% of NPs was determined by Approach I, is shown in Figure 5. The MSI data distributions are typically shown as color-scaled images at selected *m*/*z*. Here, as the presence of Ag NPs is assessed by a combination of three diagnostic ions, the MSI data were visualized in two separate images. The grayscale image in Figure 5a shows the distribution of the sum of signal intensities of ions **1**, **2**, **3**, and Ag^+^. The white color corresponds to 840 ion counts, the summed noise level of two isotopic components of the diagnostic ions; black corresponds to intensity over 10 000 ion counts and shades of gray to 840–10 000 ion counts on a linear scale. The bottom image in Figure 5b shows the combination of the detected diagnostic ions. Here, Ag^+^ ions were not shown because adding the fourth ion would increase the color code from 7 to 14, and Ag^+^ signals were detected almost exclusively in pixels containing other diagnostic ions (mostly ion **2**). Surprisingly, the data pixels containing all three diagnostic ions (olive-colored pixels) did not represent a majority of pixels with detected NPs. When comparing the two modes of display in Figure 5, it can be seen that the darkest color of the pixels in Figure 5a, representing the highest intensity, belongs to the pixel containing all three ions, while the pixels representing single ions 1, 2, or 3 have the least intense color.

**Figure 6:**
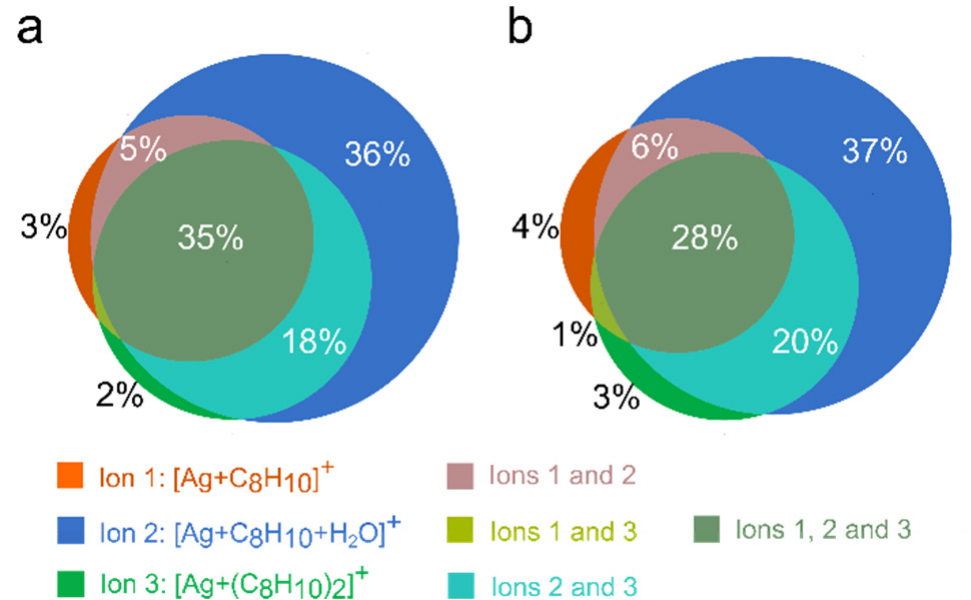
Venn diagram showing the average distribution of pixels containing a combination of diagnostic ions treated by (a) Approach I or (b) Approach II. For clarity, Ag^+^ ions were not shown here.

Figure 6 shows a Venn diagram representing the impact of diagnostic ions **1** (orange), **2** (blue), and **3** (green) on the contribution to data pixels containing them. The concurrent presence of ions **1** and **2**, ions **1** and **3**, ions **2** and **3**, and all three ions is represented as pink, light green, turquoise, and olive colors, respectively. The represented data is an average of nine spot arrays measured at three laser energy levels. The complete data obtained from these experiments, including standard deviations, are shown in Section 6, Supporting Information. It should be noted that the data pixel counts calculated using Approach I do not differ substantially from those obtained using Approach II. It is also worth mentioning that more than 90% of all data pixels contain a signal of diagnostic ion 2. There were more pixels with ion 2 solely pointing to NP presence than those with the combination of all three diagnostic ions. These observations indicate that signals of ions 1 and 3 were lost due to low signal intensity or false attribution of those to noise during mathematical data processing. Detailed data analysis of the data pixels, the absolute signals from different diagnostic ions, and intensity histograms supporting the last claim can be found in Section 7, Supporting Information.

## Conclusion

The detection of individual 80 nm Ag NPs using LDI MSI was reported for the first time. Due to the efficient gas-phase formation of complex ions of Ag with xylene and water in a subatmospheric dual LDI/ESI ion source, virtually all NPs in the sample can be detected one by one using an orbital trap analyzer.

Special attention was paid to preparing and characterizing the sample with dispersed individual NPs. An on-demand piezoelectric dispenser driven by an *XY* position table was employed to deposit droplets containing only a few NPs on the glass slides. Detailed examination of the deposited spots with SEM allowed precise counting of NPs in 50 spots of 2.7 × 1.2 mm array; the total number of NPs was 308 ± 15.

Two data processing strategies were employed: a stricter Approach I and a looser Approach II. According to the first approach, an NP is present on the pixel only if ions with two main Ag isotopes are detected, while detecting only a single Ag isotope is enough in the case of the second approach. The first approach can lead to underestimating NP numbers, while false-positive pixels could be expected when MSI data is treated by Approach II. The respective detection efficiencies were 84 and 95%. The obtained high detection efficiency of individual NPs shows the potential of the technique for bioapplications where NPs can be used as tags and would be a topic of further investigation.

In the presented case, the limiting factors were the detection limits attained in the Orbitrap mass analyzer, laser fluence, and signal processing software. It is well-known that sets of raw data produced by transient signals during imaging experiments recorded in the Orbitrap mass analyzer are larger, and to find the optimum between data size and preservation of complete information, the detection of all weak signals was not a primary focus. Thermo Extended Software Parameters license allows controlling the instrumental noise level, which may further increase the sensitivity of the technique in the future. Still, even with standard data processing methods, it was possible to detect single NPs. These results suggest that the use of the latest mass spectrometers with improved sensitivity and custom-tailored data acquisition systems (for example, FTMS Booster X2 for Orbitrap transient acquisition from Spectroswiss, Switzerland) should allow the detection of NPs with 100% efficiency. Another option of increasing the sensitivity of NP detection could be the utilization of sensitive time-of-flight or linear ion trap mass analyzers able to count single ions produced by LDI in combination with a subAP MALDI source.

## Supporting Information Available

Experimental details about the piezoelectric deposition system with examples of prepared spot arrays, an experiment showing the enhancement of the xylene addition, results showing the properties of the used laser, detailed comment on probability estimates, and, finally, detailed information on ion distribution taken from all obtained experimental results presented in the manuscript (PDF)
