# Supplementary material for: Detection of Single Ag Nanoparticles Using Laser Desorption/Ionization Mass Spectrometry
Source: J Am Soc Mass Spectrom. 2023 Jun 12;34(7):1459–66. doi: 10.1021/jasms.3c00137 (PMC10326916; doi:10.1021/jasms.3c00137)
Supplement: Supplementary file 1 — js3c00137_si_001.pdf [file js3c00137_si_001.pdf]

Supporting Information

Detection of Single Ag Nanoparticles Using Laser Desorption/ Ionization Mass Spectrometry

Michal Žalud, Vadym Prysiazhnyi, Antonín Bednařík, Jan Preisler*

Department of Chemistry, Faculty of Science, Masaryk University, 625 00 Brno, Czech Republic

Contents

1. Deposition of Ag NPs using a piezoelectric dispenser s2
2. Examples of the prepared spot arrays s3
3. Signal enhancement due to gas-phase formation of complex ions s4-s5
4. Probability estimation details s6-s7
5. Laser spot, laser energy, and energy absorbed by NPs s8-s9
6. The relative contribution of diagnostic ions in MSI data pixels s10
7. Limiting factors of Ag NP detection and intensity histograms of diagnostic ions s11-s13

1. Deposition of Ag NPs using a piezoelectric dispenser

To prepare dispersed individual Ag NPs, a drop-on-demand piezoelectric dispenser connected to the *XYZ* position table was employed. Figure S1 shows a schematic view of the experimental setup and driving voltage pulse. The driving pulse and the deposition of spot arrays (*XY* positions, number of droplets) were controlled by software developed in the LabVIEW environment. The deposition of control samples was done on Si substrate, followed by three glass slides (sample for MSI) and one more control Si substrate. The control substrates were used to verify suspension stability in time and count NPs deposited in the spot array. The control Si substrates contained 4 spot arrays, and 42 spot arrays were deposited on a single glass. The duration of a single spot array deposition was ~1.2 min. Therefore, a deposition on three glass slides and two control Si substrates took ~3 h (also including the time necessary for sample replacement in the deposition system).

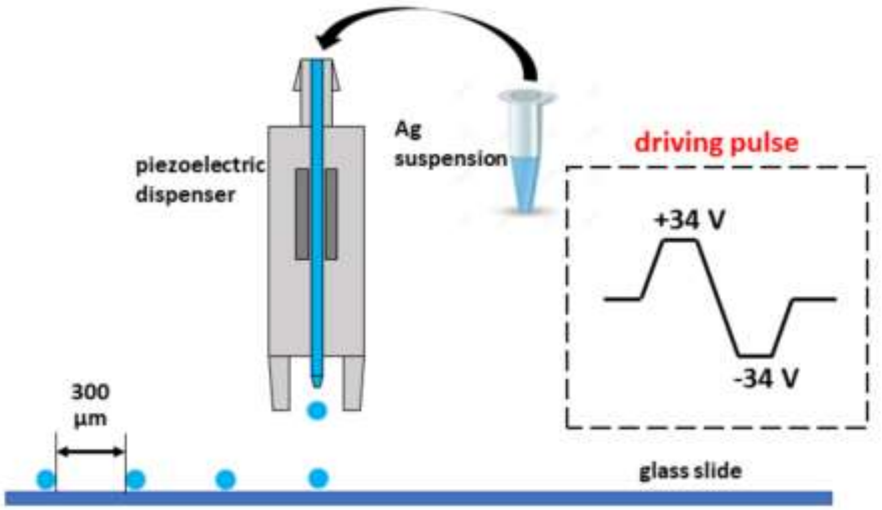

Figure S1. A schematic representation of the drop-on-demand piezoelectric dispensing system.

2. Examples of the prepared spot arrays

Figure S3 shows examples of two 10 × 5 spot arrays with 300 μm spacing deposited on Si substrate using the drop-on-demand piezoelectric dispenser. Both images were obtained during separate deposition sessions and measured on different days, demonstrating the reproducibility of the technique. It is necessary to note that the dried spot size can vary for Si substrate from 5 μm to 20 μm, and certain dislocations of the spots can be observed. Note that the white spots in the SEM image are not single NPs, but dried spots. The contrast, in this case, is coming from non-conductive crystals mostly.

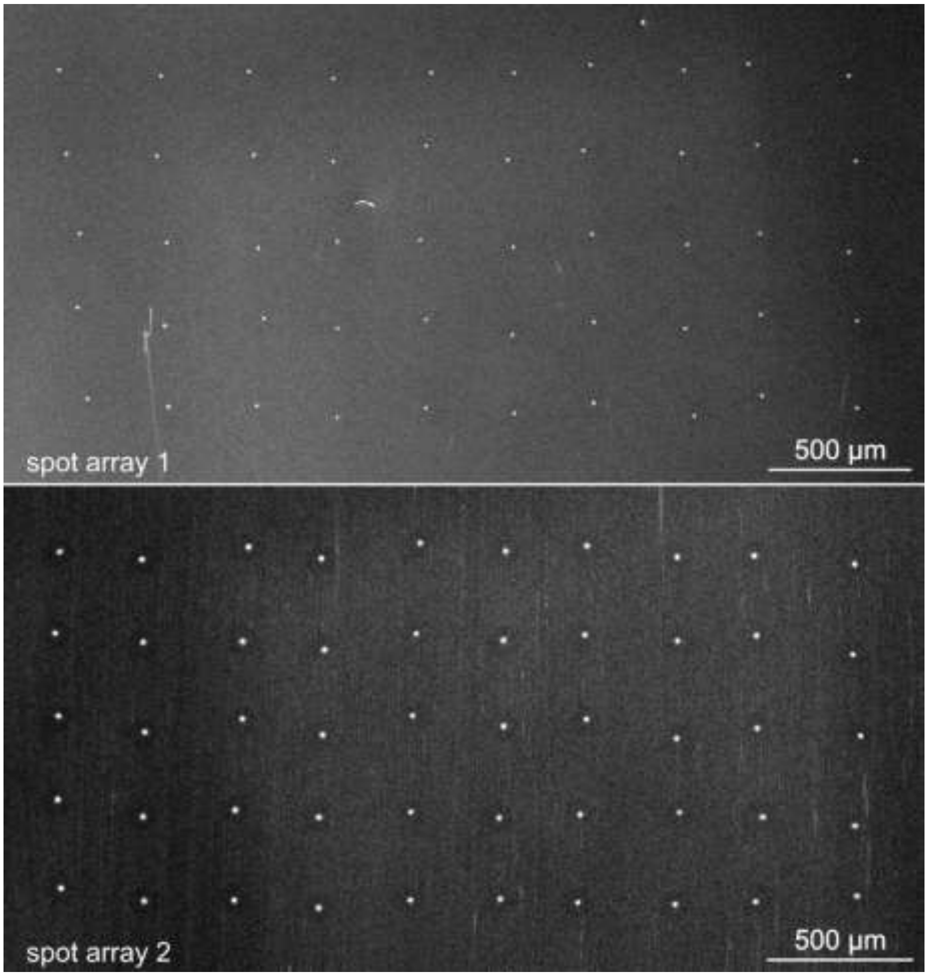

Figure S2. Examples of two 10 × 5 spot arrays deposited at the same conditions using NP suspensions prepared in different experiments.

3. Signal enhancement due to gas-phase formation of complex ions

A simple experiment was executed to demonstrate the signal enhancement by xylene vapors. A 0.5 µL of as-received Ag NP suspension was placed on the Si substrate using a standard micropipette. Based on the NP concentration ($8.1 \cdot 10^9$ NP/mL) provided by the manufacturer, the droplet should contain approximately ~$4 \cdot 10^6$ NPs. After drying, an average spot with ~300 μm in diameter was formed. Approximately half of the spot was irradiated by laser under the same conditions used for MSI measurements: pixel width 10 μm (determined by ion trap injection time) and pixel height 10 μm (defined by the distance between the adjacent scan lines). The first half of the droplet was measured with the ESI capillary open, allowing the influx of laboratory air, while the second half was measured with a 20 mL beaker with 5 mL of xylene placed close by the capillary inlet. Other details are similar to those reported before.[1] Figure S2a shows the schematic of the experiment. Figure S2b shows mass spectra averaged over a single scan line for air influx (upper graph, rose line) and xylene vapors influx (bottom graph, navy line).

The mass spectrum contained Ag ions (Ag^+, Ag_2^+, and Ag_3^+) and charged adducts when the ESI capillary inlet was exposed to laboratory air. Adding a beaker with liquid xylene and enclosing the space close to the capillary led to a formation of charged adducts of Ag and xylene, with the three most prominent being $[Ag+C_8H_{10}]^+$, $[Ag+C_8H_{10}+H_2O]^+$, and $[Ag+2(C_8H_{10})]^+$. Those are denoted in the manuscript as **1**, **2**, and **3**, respectively.

First, the most intense ion signal was compared: Ag^+ ions in the case of air influx and $[Ag+C_8H_{10}+H_2O]^+$ in the case of xylene vapors influx. The intensity was increased from $(3.3 \pm 0.8) \cdot 10^5$ ion counts to $(4.4 \pm 0.8) \cdot 10^5$ ion counts. The difference in signal intensity became more pronounced for the sum of diagnostic ions: three Ag^+ ions ($Ag^+:Ag_2^+:Ag_3^+$ as 89:9:2) compared to Ag^+ and three Ag/xylene adducts ($Ag^+:[Ag+C_8H_{10}]^+:[Ag+C_8H_{10}+H_2O]^+:[Ag+2(C_8H_{10})]^+$ as 10:27:56:8). It increased from $(1.9 \pm 0.5) \cdot 10^7$ ion counts to $(4.4 \pm 1.1) \cdot 10^7$ ion counts. As a result, adding xylene vapors improves the overall sensitivity of subAP LDI MS/MSI.

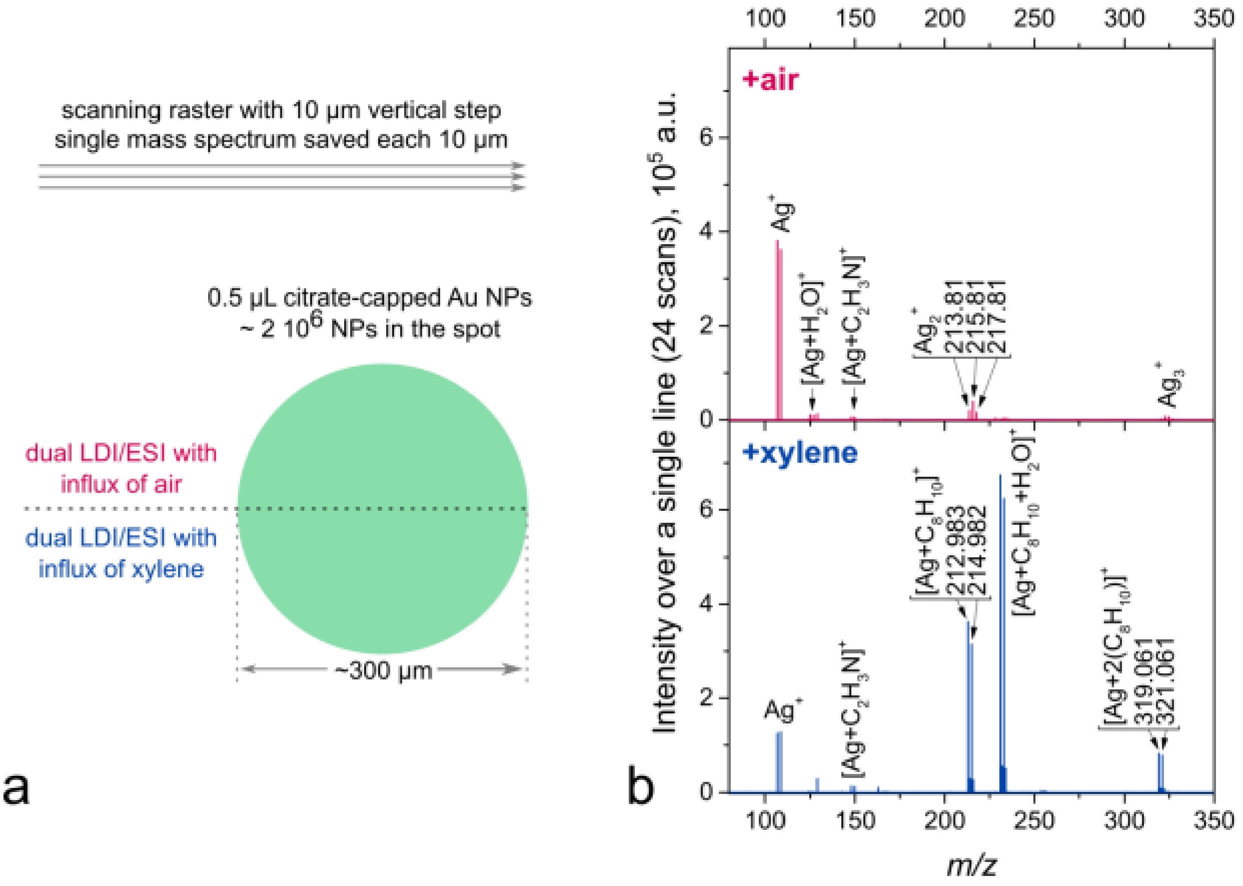

Figure S3. a) Schema of the model experiment where a spot containing ~4·10^6 NPs was irradiated with air or xylene vapor influx through the ESI capillary; b) the respective mass spectra demonstrating the ion signal increase.

4. Probability estimation details

As mentioned in the main text, two probabilities were estimated: (**1**) the occurrence of two or three NPs in a single pixel and (**2**) the location of an NP just in between two adjacent pixels; thus, a single NP can generate a signal from two adjacent pixels.

1. This estimation was based on probability (P) calculations based on the Poisson distribution of particles in the spot array. The parameters necessary for this estimation are the area, the pixel size, and the total NP number. The probability is based on the equation:

$$P_{\lambda,k} = e^{-\lambda}\frac{\lambda^k}{k!},$$

where λ is the average number of particles per pixel and k is the number of particles in one pixel (it takes values 0, 1, 2, *etc.*). The number of particles per pixel can be calculated as the total NP number, 308 in this case, divided by the total number of pixels. Only the area of 50 circular spots was counted to find the total pixel count because NPs were located only in these spots, not in the entire array area. To estimate the spot diameter on the glass substrate, the deposited single spots were sputtered by 10 nm Cu film using the same sputtering equipment and analyzed using SEM. Based on those images, a single droplet leaves a spot with a diameter of ~300 μm on a glass substrate. Thus, the total area of 50 spots in which NPs can be placed corresponds to 34990 pixels. The probability of two NPs being in a single pixel is 0.0038% pixel, and the occurrence of three NPs is 0.00001%. If each NP generates a detectable signal, it should be recorded from 307 pixels. The probability that a signal from a pixel was generated by two or three NPs is given by the ratio $P_{\lambda=2,k}/P_{\lambda=1,k}$ or $P_{\lambda=3,k}/P_{\lambda=1,k}$, respectively. These relative probabilities are 0.43% and 0.01%, meaning that two NPs can be expected only in one of 308 pixels. Note that this estimation assumes a random distribution of NPs within the spots and the absence of NP aggregation in the suspension.

2. This estimation is based on a simple assumption that a signal from an NP is generated after irradiation by the right edge of the laser spot. The signal from an 80 nm NP can be detected from both pixels only if the NP is located within an 80 nm wide strip between two pixels depicted in Figure S4. As NP can be located in any position of the pixel, the probability of signal generation

can be expressed as the ratio between the strip and pixel areas (10 × 0.04 μm) / (10 × 9.96 μm) = 0.4%. Furthermore, displacement of an NP from the pixel border below 40 nm may result in the signal dropping below the detection level. Therefore, this is an improbable event that will not be considered.

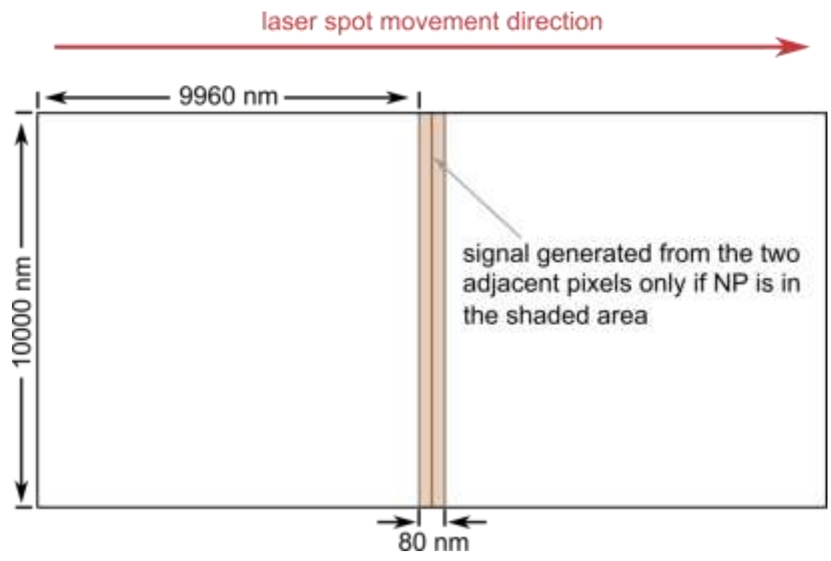

Figure S4. A scheme depicting the probability of detecting a single NP in two adjacent pixels.

5. Laser spot, laser energy, and energy absorbed by NPs

The laser spot size was estimated as an ablated area of a thin film irradiated by laser the same way as during the MSI experiments. Figure S5 shows an example of the laser spot profile at 0.55 μJ/pulse laser energy. The laser profile is not square; it is clear that the radiation profile is not homogeneous. The relative energy influx transferred to an NP at the three laser energy levels based on laser spot size, laser frequency, and sample scan speed (38.2 μm/s) is presented in Table S1. To compensate for the elliptical shape of the laser profile, the average scan length, *i.e.*, the distance where the laser spot was over the NP, was taken as 70% of the laser profile horizontal diameter as a reasonable estimation as it is clear that an NP located close to the pixel vertical center receives a higher radiation dose than the ones located at the pixel poles. The relative energy influx was calculated as energy per pulse multiplied by the number of pulses (defined by average scan length and NP irradiation duration). Note that the relative energy influx was normalized to the lower value for clarity.

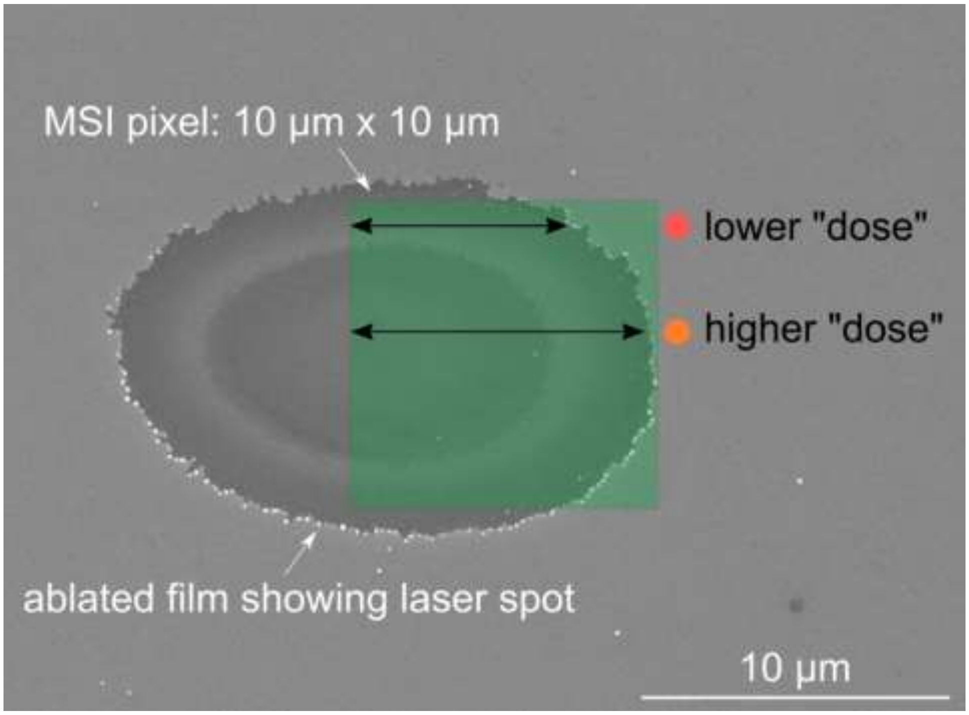

Figure S5. SEM image of laser profile together with MSI pixel size (green square) showing the difference of received laser radiation “dose” depending on the vertical position of NP relative to the pixel center.

Table S1. The number of laser pulses and relative energy influx received by an average Ag NP irradiated at four laser energy levels. The laser spot horizontal diameter was measured within a 10% error.

Energy per pulse, µJ/pulse	0.31	0.41	0.55
Horizontal diameter of the laser profile, μm	15.8	17.0	17.9
Average scan length, μm	11.1	11.9	12.5
NP irradiation duration, s	0.292	0.314	0.331
**Relative energy influx**	**1x**	**1.4x**	**2x**

6. The relative contribution of diagnostic ions in MSI data pixels

Tables S2 and S3 show percentages of pixels and the impact of ions averaged for different laser energy and averaged from the whole dataset by Approaches 1 and 2, respectively.

Table S2. Averaged data pixel fraction according to diagnostic ion contributions for different laser energies. The raw data was processed by Approach I.

Laser energy per pulse, μJ/pulse	Percentage of pixels containing specific ion(s) ± standard deviation						
	ion 1, %	ion 2, %	ion 3, %	ions 1+2, %	ions 1+3, %	ions 2+3, %	ions 1+2+3, %
0.31	4±1	52±16	0%	7±1	0%	11±6	26±10
0.41	3±3	23±5	2±1	3±2	1±1	27±6	40±7
0.55	2±1	32±10	4±4	5±3	0±1	17±7	40±3
**Average**	**3±2**	**36±10**	**2±2**	**5±2**	**0±1**	**18±6**	**35±7**

Table S3. Averaged data pixel fraction according to diagnostic ion contributions for different laser energies. The raw data was processed by Approach II.

Laser energy per pulse, μJ/pulse	Percentage of pixels containing specific ion(s) ± standard deviation						
	ion 1, %	ion 2, %	ion 3, %	ions 1+2, %	ions 1+3, %	ions 2+3, %	ions 1+2+3, %
0.31	5±1	52±14	0%	8±3	0%	15±5	20±8
0.41	4±3	26±7	4±2	5±3	2±2	28±5	32±6
0.55	4±1	33±8	6±6	5±3	1±1	18±6	33±2
**Average**	**4±2**	**37±10**	**3±3**	**6±3**	**1±1**	**20±5**	**28±5**

7. Limiting factors of Ag NP detection and intensity histograms of diagnostic ions

Here, we explore what limits the NP detection in subAP LDI MSI derived from our experiments with Ag NPs. Figure 4 shows that the probability of detecting the diagnostic ions increases with laser energy; the ions have to be generated in quantities exceeding the detection limit of the mass analyzer. Note that experiments were not carried out using the latest model of the Orbitrap mass analyzer family; therefore, we expect that using a newer instrument with higher sensitivity would lead to higher NP detection efficiency and allow deduction of the NP size. To extend the statistical analysis, signals measured from nine spot arrays were combined. It was decided to pick 3 spot arrays irradiated by 0.31, 0.41, and 0.55 µJ/pulse laser energies, resulting in a total of 1725 data pixels. Those pixels were subdivided into two groups: 1) pixels with intensity above 2520 ion counts (3× noise, recalled as "high" intensity) and with intensity from noise to 2520 ion counts (recalled as "low" intensity). This threshold between "high" and "low" intensity signals does not have particular reasoning but helps reveal differences in detected ions for low- and high-intensity signals. Table S4 answers some questions about the distributions of data pixels depending on the constraints of the chosen diagnostic Ag-xylene ions. S_n means the signal intensity of component *n*, where n = **1**, **2**, **3**, **sum** (equivalent to $S_1+S_2+S_3$). N represents the noise (840 ion counts for the sum of the isotopic components of the diagnostic ions), $\%_{total}$ is the percentage of the total number of data pixels (1725), and $\%_{high}$, $\%_{low}$ is the percentage from 1035 pixels for signals denoted as „high intensity" and 690 pixels denoted as „low intensity," respectively. The table represents answers to the questions below.

1) ***What are the individual contributions of the diagnostic ions (lines 2-4)?*** Diagnostic ion **2** occurs most frequently in the data pixels. It could be expected because it is most frequently the dominant ion, but the relative abundances of ions **1** and **3** do not follow those of ions measured for Ag film (diagnostic ions **1**:**2**:**3** as 30:62:8, respectively).
2) ***What is the influence of diagnostic ion 2 on the total intensity (lines 5-6)?*** When measured signals of the diagnostic ions have high intensity, the ratio between them is constant, as written above, while some data are lost due to the signal processing in the case of lower signals. In this case, the contribution of signal from ion **2** on the total sum obtained from Ag film should be 62% (S_2:S_{sum}=62:100 for Ag film). As expected, signals with lower intensity are often false-attributed to being a noise. The signal of ion **2** contributed over

90% of the total ion signal in 21% and 2% of data pixels with low and high intensity, respectively.

3) ***How many data pixels contain all three diagnostic ions (line 7)?*** The outcome was unexpected, as only 27% of data pixels contained signals of three diagnostic ions. Here the effect of losing data is evident: 45% of pixels with signals of all three ions are the ones with higher intensity, while pixels with a low signal intensity never contained signals of all three ions.

Table S4. Counts of data pixels within certain limiting constraints from a 1725 data pixel pool.

condition	all data pixels		S_{sum}>2540 ion counts			$N<S_{sum}\leq$2540 ion counts		
	counts	$\%_{total}$	counts	$\%_{total}$	$\%_{high}$	counts	$\%_{total}$	$\%_{low}$
1. $S_{sum}>N$	1725	*100%*	1035	*60%*	*100%*	690	*40%*	*100%*
2. $S_1>N$	648	*38%*	581	*34%*	*56%*	67	*4%*	*10%*
3. $S_2>N$	1445	*84%*	1014	*59%*	*98%*	431	*25%*	*62%*
4. $S_3>N$	861	*50%*	797	*46%*	*77%*	64	*4%*	*10%*
5. $S_2>0.6\cdot S_{sum}$	969	*56%*	496	*29%*	*48%*	473	*27%*	*69%*
6. $S_2>0.9\cdot S_{sum}$	381	*22%*	20	*2%*	*1%*	361	*21%*	*52%*
7. $S_1,S_2,S_3>N$	469	*27%*	469	*27%*	*45%*	0	*0%*	*0%*

The division of data pixels into two groups with high and low intensity was done intentionally to demonstrate that signals with low intensity tend to be lost during data processing of the transient signal. This was demonstrated on diagnostic ions **1** and **3**. The intensity histograms for the diagnostics ions plotted from the same 1725 data pixels are shown in Figure S6. Each row in the figure shows the intensity distribution for given laser energy: the bottom one for 0.31 µJ/pulse (pink colored), the middle one for 0.41 µJ/pulse (green colored), and the top one for 0.55 µJ/pulse (dark-blue colored). Each column defines from which ions the signal was obtained: the sum of ions **1**, **2**, and **3**; ion **1**; ion **2**; and ion **3** (from left to right). The bin width is set to 840 ion counts. In an ideal case, this histogram is expected to have a profile similar to Maxwell–Boltzmann distribution, but its lower part is cut due to the non-zero detection limit of the mass analyzer. The increase in energy results in a shift of maxima and the appearance of higher-intensity signals. Even for the highest energy input, the intensity distributions of ions **1** and **3** differ from the one for ion

2. Nevertheless, even with those well-known limitations, detecting signals from individual NPs is still possible.

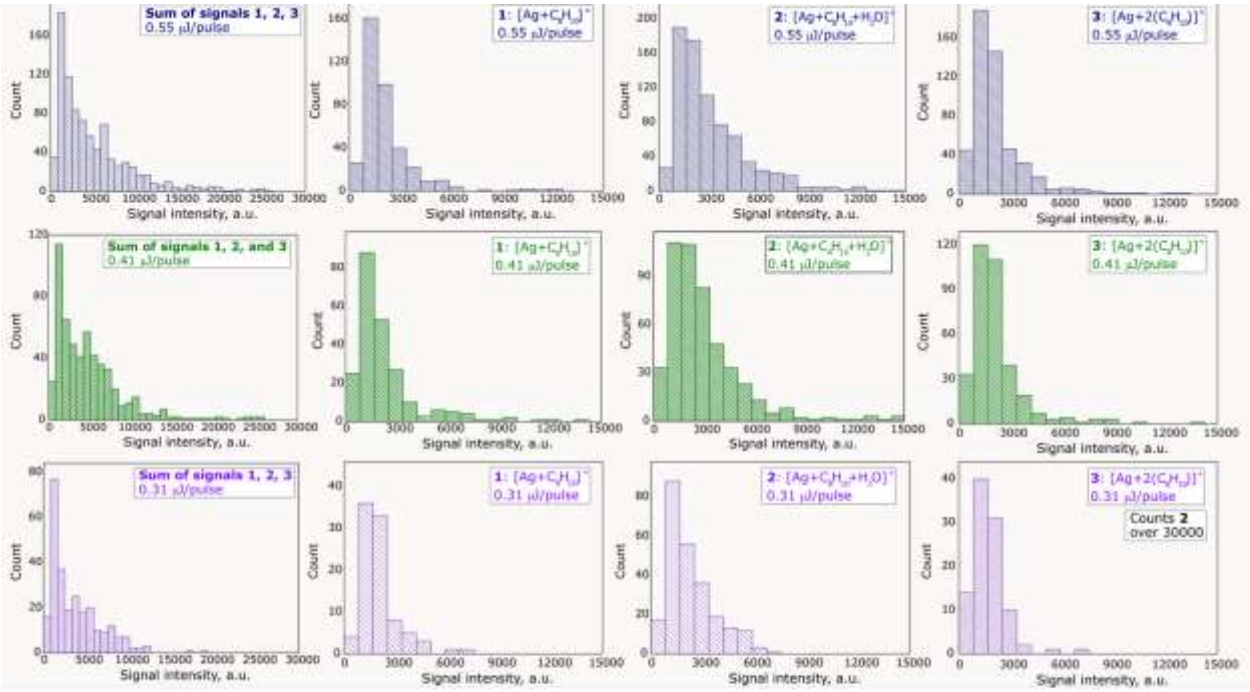

Figure S6. Intensity distribution diagrams for 1725 data pixels obtained from 9 spot arrays after MSI measurements. Each row corresponds to laser energy per pulse values (from bottom to top: 0.31, 0.41, and 0.55), and each column represents a specific type of ion (from left to right: the total sum of ions **1**, **2**, and **3**; only ion **1**, only ion **2**, only ion **3**).

References

(1) Bednařík, A.; Prysiazhnyi, V.; Preisler, J. Metal Ionization in Sub-Atmospheric Pressure MALDI Interface: A New Tool for Mass Spectrometry of Volatile Organic Compounds. *Anal. Chem.* **2021**, *93* (27), 9445–9453. https://doi.org/10.1021/acs.analchem.1c01124
